# Medical and financial burden of acute intermittent porphyria

**DOI:** 10.1007/s10545-018-0178-z

**Published:** 2018-04-19

**Authors:** Rochus A. Neeleman, Margreet A. E. M. Wagenmakers, Rita H. Koole-Lesuis, G. Sophie Mijnhout, J. H. Paul Wilson, Edith C. H. Friesema, Janneke G. Langendonk

**Affiliations:** 1 Porphyria Center, Center for Lysosomal and Metabolic Disease, Department of Internal Medicine, Erasmus Medical Center, PO Box 2040, 3000 CA Rotterdam, the Netherlands; 2 Department of Internal Medicine, Isala Clinics, Zwolle, the Netherlands

**Keywords:** Acute porphyrias, Acute intermittent porphyria, Porphyria, acute intermittent/epidemiology, Heme arginate, Nervous system diseases

## Abstract

**Introduction:**

A small proportion of patients with acute intermittent porphyria (AIP) suffer from recurrent porphyric attacks, with a severely diminished quality of life. In this retrospective case-control study, the burden of disease is quantified and compared among three AIP patient subgroups: cases with recurrent attacks, cases with one or occasional attacks and asymptomatic carriers.

**Methods:**

Data from patient records and questionnaires were collected in patients between 1960 and 2016 at the Erasmus Medical Center, Rotterdam, the Netherlands. We collected symptoms related to porphyria, porphyria related complications, attack frequency, hospitalisation frequency, hospitalisation days related to acute porphyric attacks, frequency of heme infusions and medical healthcare costs based on hospitalisations and heme therapy.

**Results:**

In total 11 recurrent AIP cases, 24 symptomatic AIP cases and 53 AIP carriers as controls were included. All recurrent patients reported porphyria related symptoms, such as pain, neurological and/or psychiatric disorders, and nearly all developed complications, such as hypertension and chronic kidney disease. In the recurrent cases group, the median lifelong number of hospitalisation days related to porphyric attacks was 82 days per patient (range 10–374), and they spent a median of 346 days (range 34–945) at a day-care facility for prophylactic heme therapy; total follow-up time was 243 person-years (PYRS). In the symptomatic non-recurrent group the median lifelong number of hospitalisation days related to porphyric attacks was 7 days per patient (range 1–78), total follow-up time was 528 PYRS. The calculated total medical healthcare cost for recurrent cases group was €5.8 million versus €0.3 million for the symptomatic cases group.

**Electronic supplementary material:**

The online version of this article (10.1007/s10545-018-0178-z) contains supplementary material, which is available to authorized users.

## Introduction

Acute intermittent porphyria (AIP) is a rare inherited metabolic disorder in which patients suffer from acute porphyric attacks with abdominal pain, often with anxiety, nausea, tachycardia and hypertension. During severe attacks, psychiatric symptoms, hyponatremia, seizures and progressive peripheral neuropathy can occur (Bylesjö et al 2009). Such attacks can progress to paralysis, respiratory failure, and death if patients are not treated. Apart from acute porphyric attacks, patients have a long term increased incidence of hypertension, kidney disease and hepatocellular carcinoma (HCC) (Andersson et al 1996; Andersson et al 2000; Sardh et al 2013).

AIP is caused by mutations in the hydroxymethylbilane synthase (*HMBS*, OMIM 609806) gene. In literature it has been described that less than 10% of *HMBS* gene mutation carriers experience acute porphyric attacks during their lives (Puy et al 2010). However, a recent study indicates an even lower clinical penetrance of about 1% (Chen et al 2016). Ever since heme became the treatment of choice in the 1980s, porphyria attacks have been treated with intravenous heme. This has been shown to be effective in improving both the biochemical abnormalities, clinical symptoms and preventing deterioration of the neurological defects. It is therefore regarded as a life-saving therapy (Whatley and Badminton 2013).

Only a small subset of the symptomatic patient group suffers from recurring porphyric attacks several times per year. Recurring attacks are treated with weekly prophylactic heme infusions (Marsden et al 2015). This therapy is, however, complicated by iron overload, phlebitis, venous thrombosis and frequent need for venous access devices, and is a significant burden for the patient (Willandt et al 2016). Patients who suffer from recurrent attacks report a low quality of life (QoL) and a negative impact on several aspects of everyday life, including unemployment, personal relationships and long-term disability (Millward et al 2001; Jiménez-Monreal et al 2015; Naik et al 2016).

New in this study is the analysis of the recurrent patients separate from sporadically symptomatic cases, and *HMBS* gene carriers as controls. Although recurring porphyric attacks have been shown to be associated with a poor quality of life, the financial and personal burden of this disease in relation to the occurrence and frequency of attacks are unclear. Therefore, in order to get a better insight into the burden of AIP, we studied the prevalence of porphyria symptoms and complications, in addition to the costs related to recurrent porphyric attacks and compared them to symptomatic porphyria cases and asymptomatic gene carriers in a case-control study design.

## Methods

### Data collection

In this single-centre retrospective longitudinal observational cohort study, all patients with AIP attending the clinic of the Porphyria Center in the Erasmus Medical Center (Rotterdam, the Netherlands) between 1960 and 2016 were included. The diagnosis AIP was based on either a known *HMBS* gene mutation, or on positive biochemistry (defined as a delta-aminolaevulinic acid (ALA) and porphobilinogen (PBG) level at least four times the upper limit of normal) in combination with a decreased porphobilinogen deaminase (PBGd) enzyme activity measured in either erythrocytes or lymphocytes.

Data were collected by R.A.N. and J.G.L. from patients’ electronic and paper records, and from self-reported questionnaires (Suppl. file 1). The patient charts and file data were first reviewed. When the retrospective data was unclear (e.g. due to old, poorly hand-written notes), a consensus was formed which was entered in the final database, or the data was discarded.

AIP patients with a proven diagnosis were eligible for inclusion. A confirmed acute porphyric attack was defined as an episode of abdominal pain in parallel with a significant rise in urinary ALA and PBG levels (≥ 4 times upper limit of normal) which necessitated a verified visit or admission to a hospital for diagnosis and treatment. Hospitalisations were defined as hospitalisations with a confirmed acute porphyric attack.

We collected data on (1) porphyria symptoms and long-term complications, (2) employment status, (3) hospitalisation episodes, hospitalisation days, amount and frequency of heme infusions (heme arginate, Normosang®, Orphan Europe), (4) heme dosage schedules, duration of prophylactic heme therapy, complications of heme therapy, placement of a Port-A-Cath® (PAC, Smiths Medical), oral contraception use, therapy with GnRH analogues, analgesia dependence and the age and cause of death. The annualised attack rate was defined as the number of attacks in all observed years of follow up, counting the number of confirmed acute porphyric attacks and dividing by the number of follow up years.

The medical ethics committee of the Erasmus Medical Center, Rotterdam, the Netherlands, approved prospective and retrospective data collection for this study (MEC 2014–593). Patients were asked to sign informed consent during visits to the outpatient clinic. Also, they have approved the use of anonymous retrospective medical data without informed consent.

### Patient subgroup definitions

AIP patients were classified in three subgroups based on phenotypic characteristics. The first subgroup comprised cases with recurrent attacks, defined as patients who have more than four attacks in any year, or are on prophylactic heme therapy. The second subgroup was comprised of symptomatic cases, defined as patients who have experienced at least one confirmed acute porphyric attack, but who did not meet the definition of recurrent attack cases. The third subgroup comprised asymptomatic controls, defined as persons who carry a *HMBS* gene mutation but have never experienced a proven acute porphyric attack.

### Cost of care analysis

To calculate the costs of care, we examined paper or electronic hospital records to count the number of verifiable heme infusions and hospitalisations (for day care and regular admission) for each individual. The approach was to be as conservative as possible, all data needed to be verifiable in the charts. Total basic-costs were calculated per individual including only hospitalisations, use of day-care facilities and heme arginate ampoules; the costs are presented per group. Prices are calculated based on 2016 data, historical prices or inflation were not taken into account. These basic costs of care are therefore limited to costs of hospital care and medication and do not take other indirect costs to the patient or society into account.

### Statistical analysis

Statistical analyses were performed with IBM SPSS Statistics for Windows (version 21.0. Armonk, NY: IBM Corp.). Parameters were tested for normality using the Shapiro-Wilk test for normality. Normally distributed numerical data were compared among groups with one-way ANOVA, Bonferroni corrected. Non-parametric numerical independent data were analysed with Kruskal-Wallis’ test. Categorical data were analysed using the linear-by-linear Chi-square association-test, or Fischer’s exact test.

### Supplementary methods description

Assays for porphyrins, porphyrin precursors and enzyme activity measurements were analysed as previously described (de Rooij et al 2003), see Suppl. file 2.

## Results

### Cohort description

The study cohort consisted of 88 individuals with AIP from 49 families, baseline characteristics are presented in Table 1. In 77 patients the diagnosis of AIP was confirmed by identifying a *HMBS* mutation. Twenty-five different *HMBS* gene mutations were identified (Suppl. file 3), the R116W (c.346C>T) mutation was the most common mutation (21 subjects, 24%). In the remaining 11 individuals the diagnosis AIP was based on biochemical test results in combination with decreased HMBS enzyme activity.

**Table 1:** Baseline characteristics of acute intermittent porphyria cohort, categorised in three groups based on symptoms

	Recurrent cases (*n* = 11)	Symptomatic cases (*n* = 24)	Asymptomatic controls (*n* = 53)	All (*n* = 88)
Attacks – lifelong (*n*)	8 (2–54)	2 (1–7)	0	
Sex (% female)	63.6	75.0	66.0	68.2
BMI ^a^ (kg/m^2^) (mean ± SD)	24.9 ± 6.2	25.1 ± 8.1	25.2 ± 4.4	25.1 ± 5.8
Age at diagnosis (years)	31 (14–55)	30 (2–52)	32 (5–72)	31 (2–72)
Age of onset (years)	36 (16–56)	30 (18–55)	–	30 (16–56)
Smoking (*n*, % ever smoker)	*6*, 55	*11*, 46	*18*, 34	*35*, 40
*HMBS* gene mutations (*n*)	5	16	18	25
No. of families within group (*n*)	11	20	33	49
Follow-up duration (PYRS)	21 (3–38)	22.5 (0–53)	8 (1–45)	13 (0–53)

Data are presented with median (range), unless otherwise specified. There was no statistical difference between groups for sex, BMI, age of diagnosis and age of onset

Attack numbers were based on life-long verified acute porphyric attacks requiring hospitalisation. Subgroups were based on phenotypic characteristics: recurrent cases were defined as having more than four attacks in any year, or on prophylactic heme therapy; symptomatic cases were defined as having experienced one or more confirmed acute porphyric attack; asymptomatic controls never experienced a proven acute porphyric attack. Data on smoking are presented as life-long prevalence

BMI, body mass index; HMBS, hydroxymethylbilane synthase; PYRS, person-years

^a^first available BMI after age ≥ 18 years

**Fig. 1:**
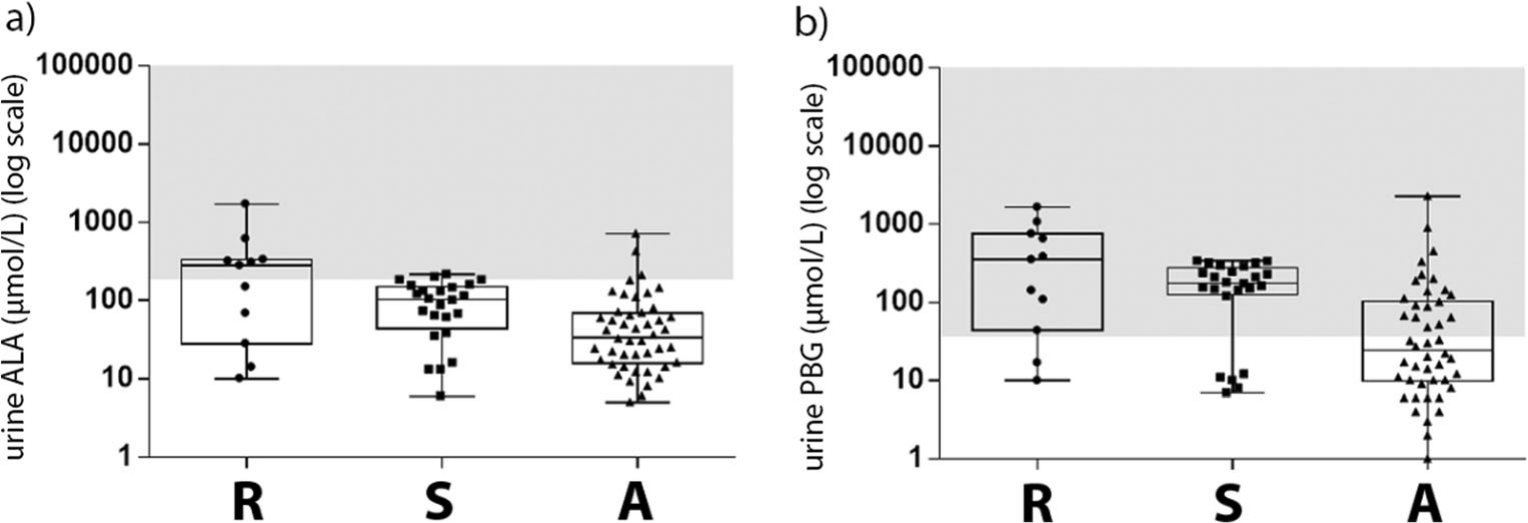
The distribution of first measured urinary porphyrin precursors in the different AIP groups. Subgroups were based on phenotypic characteristics: recurrent cases were defined as having more than four attacks in any year, or on prophylactic heme therapy; symptomatic cases, were defined as having experienced one or more confirmed acute porphyric attack; asymptomatic controls never experienced a proven acute porphyric attack. Abbreviations: *A*, asymptomatic controls; *ALA*, delta-aminolevulinic acid; *PBG*; porphobilinogen; *R*, recurrent cases; *S*, symptomatic cases. The grey areas mark increased levels, starting at 4 times the upper limit of normal (ULN) 4xULN ALA ≥ 184 μmol/L 4x ULN PBG ≥ 36 μmol/L

Eleven patients (13%) were categorised as recurrent cases (subgroup R), 24 (27%) patients were categorised as symptomatic cases (subgroup S) and 53 persons (60%) were categorised as asymptomatic AIP carriers (subgroup A, control group). The male-to-female ratio, BMI and age at diagnosis were similar in all three groups. The age of onset of symptoms was not different between both symptomatic groups. Laboratory results are presented in Fig. 1 and details are presented in Suppl. file 4.

### Prevalence of porphyric symptoms

Life-long prevalence of acute porphyria-related symptoms, long-term complications and other parameters such as unemployment in all three groups are presented in Table 2. Patients in the recurrent cases group report more porphyria related symptoms: pain, neurological deficits, and psychiatric symptoms. These differences were statistically significantly (*p*-values <0.001), except for the psychiatric symptoms. The differences between reported symptoms from patients in both symptomatic groups are smaller and mostly not statistically significantly different. However, the prevalence of several symptoms is at least double in the recurrent cases group compared to the symptomatic group, Suppl. file 5; malaise (*p* = 0.01, 82% of patients in recurrent group versus 29% in symptomatic group), fatigue (*p* < 0.01, 91 vs 38%), nausea (*p* < 0.01, 100 vs 46%), anxiety (*p* < 0.01, 46 vs 21%), psychosis/hallucinations (*p* = 0.03, 36 vs 4%) and seizures (*p* < 0.05, 46 vs 13%).

**Table 2:** Prevalence in percentages (%) of life long porphyria-related symptoms and complications in subgroups of the acute intermittent porphyria cohort

	Recurrent cases (*n* = 11)	Symptomatic cases (*n* = 24)	Asymptomatic controls (*n* = 53)	Linear-by-linear Chi^2^-association test
Acute symptoms^a^	100	83.8	35.8	*p* < 0.001
Pain	100	91.7	30.2	*p* < 0.001
Neurological symptoms	81.8	45.8	17.3	*p* < 0.001
Psychiatric symptoms	81.8	33.3	18.9	*p* < 0.001
Long-term complications				
Hypertension	72.7	70.8	26.4	*p* < 0.001
Chronic kidney disease	63.6	45.8	13.2	*p* < 0.001
Hepatocellular carcinoma	9.1	8.3	1.9	*p* = 0.15
Other parameters				
Anemia	63.6	16.7	5.7	*p* < 0.001
Seizures	45.5	12.5	0	*p* < 0.001
Dependence on analgesics	63.6	0	3.7	*p* < 0.001
Unemployment	63.6	33.3	20.8	*p* = 0.021

Data are presented as the percentage (%) of patients or persons scoring positive on one of the symptoms in the categories during their life. Subgroups were based on phenotypic characteristics: recurrent cases were defined as having more than four attacks in any year, or on prophylactic heme therapy; symptomatic cases were defined as having experienced one or more confirmed acute porphyric attack; asymptomatic controls never experienced a proven acute porphyric attack. Data on unemployment rate are presented as life-long prevalence.

Statistical differences between groups was tested with linear-by-linear Chi-squared association test, *p*-values are presented

In Suppl. file 5, all scored symptoms and p-values are presented

^a^Acute symptoms are: malaise, fatigue, nausea, vomiting, diarrhoea, constipation and red urine

### Prevalence of long-term complications

Hypertension was more prevalent in the recurrent cases group (73%) and in the symptomatic cases (71%) compared to the asymptomatic control group (26%). Kidney disease was also more prevalent in the recurrent cases (64%) and symptomatic cases (46%) compared to asymptomatic controls (13%), Table 2 and details in Suppl. file 5. There were four cases with hepatocellular carcinoma (HCC); one from the recurrent cases, two from the symptomatic cases, and one from the asymptomatic controls. One of the HCC patients was still alive at the time of database lock. In three patients, the tumour was inoperable and/or there were metastases at presentation. The fourth patient unfortunately died due to surgical complication, although the tumour was detected as an incidental finding in a curative stage.

Anemia was present in 14 cases, and mostly prevalent in the recurrent cases (7/11, 64%) and symptomatic cases (4/24, 17%) compared to asymptomatic controls (3/53, 6%). Kidney disease was present in 79% of the anemia cases (11/14). Anemia without renal insufficiency was present in 21% of all anaemic persons (3/14).

### Number of hospitalisations, day-care-admissions, heme therapy and attack frequency

The life-long hospital admissions, duration and the calculated costs are presented in Table 3. Individuals from the recurrent group were hospitalised for a median of 82 days and received a median of 399 total ampoules of heme per person. Heme schedules differed among patients and over time, ranging from every other week, to weekly and biweekly. Individuals from the symptomatic group were hospitalised for a median of 7 days for acute attacks and received a median of 3 ampoules of heme per person. The median total cost per person of combined heme/albumin infusions for the recurrent subgroup was € 292.000 versus €2.200 for the symptomatic subgroup. The costs for the recurrent patient group accounted for at least 95% of the total costs.

**Table 3:** Hospitalisations, day-care admissions and costs related to proven acute porphyric attacks in presented acute intermittent porphyria cohort

	Recurrent cases (*n* = 11)	Symptomatic cases (*n* = 24)
Hospitalisations days (*n*)	82 (10–374)	7 (1–78)
Day-care treatment (*n*)	346 (34–945)	0
Heme arginate therapy (*n*)	399 (79–945)	3 (0–24)
Hospitalisation costs (in k euros)	41 (5–193)	3.5 (0.4–39)
Day-care costs (in k euros)	149 (15–406)	0
Heme arginate costs (in k euros)	292 (58–692)	2.2 (0–17.5)
Follow-up time (years)	21 (3–38)	22.5 (0–53)
Total group cost (in k euros)	5843	288
Total follow-up time (PYRS)	243	528
Cost per person year (in k euros)	24 (3–63)	0.5 (0^a^ – 31)

All data are presented as: median (range), and data are given per person per group over total observation period, except for total group cost and total follow-up time in PYRS. The presented costs are an estimation, see manuscript for more details

Subgroups are based on phenotypic characteristics: recurrent cases are defined as having more than four attacks in any year, or on prophylactic heme therapy; symptomatic cases are defined as patients who have experienced at least one confirmed acute porphyric attack

Costs were calculated using prices based on 2016 for hospital stay and heme arginate.

k, 1000; PYRS, person-years

^a^One patient did have an acute porphyric attack, however, data on details were missing, and costs could not be calculated and are thus presented as zero, this patient was the *index* patient in the family

**Fig. 2:**
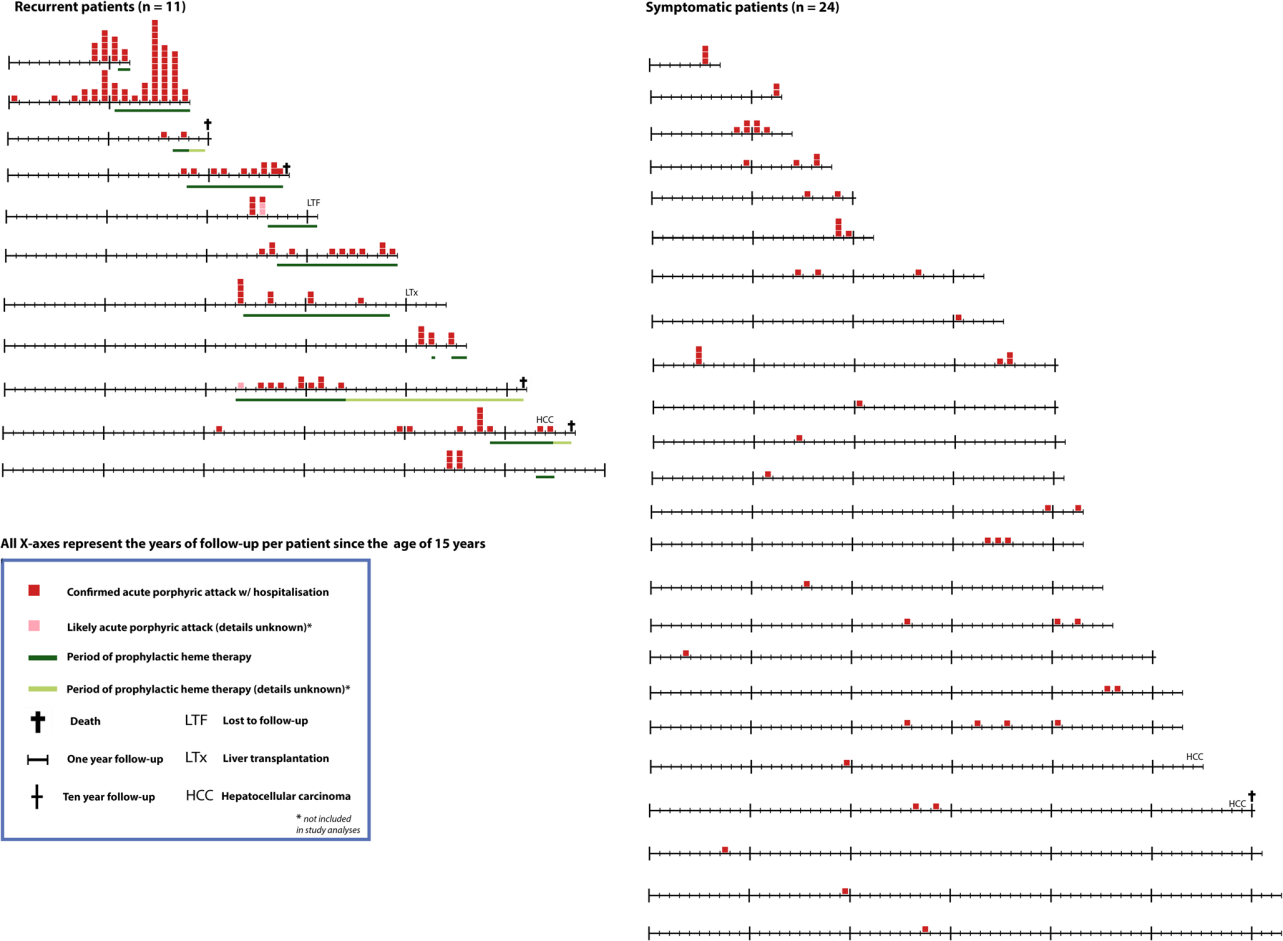
Schematic information with individual life lines with porphyric attacks of all symptomatic cases, including information regarding prophylactic heme therapy. Subgroups were based on phenotypic characteristics: recurrent cases were defined as having more than four attacks in any year, or on prophylactic heme therapy; symptomatic cases, were defined as having experienced one or more confirmed acute porphyric attack; asymptomatic controls never experienced a proven acute porphyric attack. On the left there are 11 lines derived from patients with recurrent attacks, and on the right 24 lines derived from symptomatic cases. Each line starts at 15 years of age and ends at the end of the observation period or death. Each segment on the line represents one year. The red blocks represent one attack during that year. The green bars under the timelines represent years in which patients received prophylactic heme. A cross represents death. Abbreviations. *LTF*, lost to follow-up; *HCC*, hepatocellular carcinoma; *LTx*, liver transplant. *not included in study analysis

We calculated the number of attacks for the symptomatic and recurrent groups, per year of total follow-up. In total, in the recurrent group there were 137 acute attacks in 407 years of follow-up, or 0.3366 attacks per year. In the symptomatic group, there were 56 acute attacks in 959 years of follow-up, or 0.0584 attacks per year. The patients in the recurrent group suffered 5.8 times more attacks during follow-up. Figure 2 illustrates the follow-up time, the attacks and heme therapy for each patient in the symptomatic and recurrent groups over the observation period.

Comparing the number of attacks before the start of heme prophylaxis to the number of attacks during heme prophylaxis, a 50% reduction in number of attacks was observed; before heme prophylaxis there were 2.28 attacks per year, during heme prophylaxis 1.11 attacks per year.

### Description of cases with premature death

At the end of the observation period (31 December 2016) the median age of the AIP cohort was 54 years (range 18–81). Five patients died during the observation period, median age of death was 65 years (range 34–72 years). All deaths were directly or indirectly related to porphyria. Four out of five patients were in the recurrent attack group. One person died in the symptomatic case group, this was a male patient presenting at age 41 years with a first uncomplicated porphyric attack. At age 72 years, HCC was diagnosed following an incidental finding on a PET CT-scan performed for headache. He underwent hemi-hepatectomy but died from a brainstem hemorrhage, 13 days post-operatively. A detailed description of all deceased cases is presented in Suppl. file 6.

### Recurrent cases subgroup

The details of the recurrent cases subgroup are presented in Table 4. Four out of 11 patients (36%) were male. The median number of attacks requiring hospitalisation was eight per patient (range 2–54) following a median observation time of 21 years (range 3–38). The total number of hospitalisation days per patient ranged between 10 to 374 days. Analgesic dependency was a problem in nearly all recurrent patients (82%, 9/11). Prophylactic heme therapy was initiated in a patient following only two attacks, this was deemed necessary following progression of neurological symptoms and a progressive rise in ALA and PBG levels following the first two attacks.

**Table 4:** Overview of hospitalizations, treatment, and calculated costs in the recurrent AIP patients group

**Case**	**1**	**2**	**3**	**4**	**5**	**6**	**7**	**8**	**9**	**10**	**11**
Sex	F	F	M	F	M	F	M	M	F	F	F
Age of first attack (years)	16	22	22	38	35	36	56	36	37	39	30
Deceased (age in years)			42			71			65		34
Cause of death^a^			Severe attack – respiratory failure			HCC			Unknown^b^		Euthanasia^b^
Hospitalizations (n)	54	16	11	11	11	8	7	8	6	4	2
Hospitalizations (in days)	374	185	367	248	60	40	37	33	102	20	10
Heme (ampoules)	508	95	945	890	613	168	102	90	571	399	79
Total medical costs (in 1k euros )	331.2	61.9	616.0	580.2	399.6	109.5	66.5	58.7	372.2	260.1	51.5
Observation time (months)^c^	204	36	245	420	48	432	159	456	404	252	420
Total time on prophylactic heme (months)^c^	86	14	126	171	152	71	20	14	51	27	16
PACs/AV-shunts (n)	3-4	0	3	5	2	2	0	2	4	1	0
Last measured serum ferritin (normal 10 – 140 ug/L)	1470	390	3397	1523	1075	1904	NA	37	14	638	NA

The reported hospitalizations were all for confirmed acute porphyric attacks, confirmation was given by a raise in urinary ALA&PBG at the time of presentation with abdominal pain. Presented medical costs are based on prices in 2016.

Used abbreviations. 1k, 1000; ALA, delta-aminolevulinic acid; AV-shunt, arteriovenous-shunt; F, female M, male; HCC, hepatocellular carcinoma; NA, not available PAC, port-a-cath;

^a^for details see supplementary file 5

^b^Not related to a porphyric attack

^c^until 31/12/2016 or per date of death

Prophylactic heme schedules were every other week, weekly, or biweekly, with a duration ranging from 1 to 14 years. PAC venous access catheters were required in 73% (8/11) of the cases to secure venous access. However, venous access was never an absolute limitation for heme therapy. The total number of heme infusions ranged from 79 to 945 per patient, while total lifelong medication costs ranged from €51.500 to €616.046 per patient. Reported side effects of heme therapy were increased serum ferritin levels and possibly iron overload (9/11, 82%) and phlebitis (4/11, 36%); iron overload was treated by phlebotomies.

Eleven patients on prophylactic heme therapy have made attempts to be weaned off heme therapy. This has triggered acute porphyric attacks in nine patients. One patient was weaned off heme by slowly reducing the amount of heme over a period of a year, this was followed by a liver transplantation (Willandt et al 2016), the only liver transplantation in this cohort.

Two patients were treated with hormonal interventions. One was treated with a GnRH-analogue (Syranel®, Pfizer Inc.) for a limited number of months, since she developed burdensome postmenopausal symptoms and no effect on reducing attacks. A second patient endured four porphyric attacks within 6 months, but following placement of a hormonal IUD, she has been free from attacks for 2 years now (52 mg levonorgestrel-releasing intrauterine device (IUD), Mirena®, Bayer HealthCare).

## Discussion

This study is based on a longitudinal patient cohort and aims to quantify the severity of disease in AIP patients with recurrent attacks and compares this group to patients with sporadic symptoms and asymptomatic carriers. Patients with recurrent AIP have a high mortality rate and severe morbidity. Severe physical and psychological symptoms, including temporary or permanent disability, lead to unemployment in two-thirds of the recurrent patients. Hypertension and kidney disease were observed in respectively 73 and 64% of patients with recurrent AIP disease. Frequent hospitalisations for acute porphyric attacks, and frequent hospital visits when on prophylactic heme therapy alone, have a major impact on their lives and contribute most to the total calculated medical costs.

Studies that focus specifically on the recurrent patient group are scarce and mainly focus on the group’s diminished quality of life and therapeutic interventions in patient series (Marsden et al 2015; Schulenburg-Brand et al 2017). A recent qualitative study focused on the recurrent patient group (Naik et al 2016). They investigated the patients’ disease experience and concluded recurring patients have a markedly impaired QoL and often turn to alternative medicine. Another study interviewed five recurrent attack patients and showed that all of them reported psychological and coping issues with regard to the dysfunction the recurrent attack state causes (Wikberg et al 2000). The preliminary results of the prospective ‘EXPLORE’ study (Congress abstracts: https://icpp2017.org/wp-content/uploads/2017/06/OCs.pdf) show a high burden of disease in patients with frequent attacks. Our findings are in line with these findings, and illustrate one of the effects of the poor QoL in the high level of unemployment in the recurrent group. The present study shows there is a large difference in hospitalisation frequency and subsequent medical costs between recurrent and sporadic symptomatic patients. The increased burden, together with the costs, underline the importance to differentiate among recurrent cases, symptomatic cases and asymptomatic *HMBS* gene carriers. Asymptomatic *HMBS* gene carriers are without obvious disease burden or related medical costs, and will require separate studies to detect any long-term effects of the mutation.

Major strengths of this study are the prospectively defined clear study endpoints and categorisation of patients based on strict criteria for acute porphyric attacks to make comparisons among the different AIP patient groups possible. However, by using these strict criteria, an underestimation of the true impact on patient’s life and costs is likely, which may be regarded as a limitation. Furthermore, a retrospective study design, with data collection in historical, hand-written, medical charts and hospital records, will have resulted in missing data, and again in an underestimation of the burden and healthcare costs. Since 2014, we have collected data prospectively with questionnaires which can lead to recollection bias in all patient groups. There is also a definite underrepresentation of asymptomatic *HMBS* carriers in this cohort; based on the number of AIP families. Females were overrepresented in the present cohort (68%). This overrepresentation was expected in the symptomatic groups, since females have an increased risk of attacks (Bonkovsky et al 2014). However, this cannot explain the overrepresentation in the asymptomatic group which might be caused by selection bias, since women often have other reasons for abdominal pain.

There is a high prevalence of long term complications, such as hypertension, chronic kidney disease and hepatocellular carcinoma in AIP. In the present study, an increased prevalence of hypertension and chronic kidney disease in the recurrent patient group was present, compared to the other groups. Factors contributing to the occurrence of complications have not yet been elucidated. Long-term exposure to elevated plasma levels of ALA and PBG has been postulated to play a role. However, we’ve found a marked overlap in ALA and PBG levels among the recurrent, the symptomatic cases and asymptomatic *HMBS* gene carriers. There is no good explanation why some patients with markedly increased levels are without symptoms or long-term complications. Such subjects are often referred to as “asymptomatic high excreters”. In this group, plasma ALA PBG are often as high or even higher than those found in symptomatic patients *(data not shown).* For future studies this could be an interesting group to examine. Other mechanisms may play a role in AIP-associated tissue damage, such as human peptide transporter 2 (hPEPT2) genotypes, that are associated with AIP-associated kidney damage (Tchernitchko et al 2016). Hypertension was not associated with renal function decline in AIP patients (Pallet et al 2015).

Figure 2 illustrates the effect of prophylactic heme therapy on attack rates in the individual patients. This therapy is not only expensive, but also time-consuming for the patients, and not fully successful in most patients. However, interrupting their schedules triggered severe acute porphyric attacks in nearly all patients. These rebound attacks are probably in part due to prolonged induction of hepatic heme oxygenase by administration of exogenous heme, resulting in increased heme turnover (Doberer et al 2010; Thomas et al 2016). Interestingly, it has been recently shown that the prevalence of patients with recurring attacks has increased since the introduction of heme arginate therapy (Schmitt et al 2018). An explaination could be that the prevalence of patients with recurring attacks has increased since the introduction of heme arginate therapy. There are promising emerging therapies for AIP such as gene-therapy, short interfering RNA (Chan et al 2015; Fontanellas et al 2016), which are undergoing clinical trials; until these therapies become available, heme will remain the most effective therapy. Consensus definitions of acute porphyric attacks and acute porphyric symptoms should be developed, in order to define indications for initiation and schedules, in patient with frequent and/or disabling porphyric attacks.

### Conclusion and recommendations

In conclusion, AIP patients with recurrent attacks have a high mortality and morbidity, compared to other symptomatic AIP patients and to asymptomatic carriers. Recurrent attacks result in high medical costs and high unemployment rates. In future studies, these different AIP groups should therefore be analysed separately, as our study clearly demonstrated that patients with recurrent attacks are different than sporadical symptomatic patients and asymptomatic *HMBS* gene carriers.

## Electronic supplementary material

Supplementary file 1 (DOCX 33 kb)

Supplementary file 2 (DOCX 17 kb)

Supplementary file 3 (DOCX 13 kb)

Supplementary file 4 (DOCX 17.1 kb)

Supplementary file 5 (DOCX 16 kb)

Supplementary file 6 (DOCX 13 kb)
