# Supplementary material for: Medical and financial burden of acute intermittent porphyria
Source: J Inherit Metab Dis. 2018 Apr 19;41(5):809–17. doi: 10.1007/s10545-018-0178-z (PMC6133185; doi:10.1007/s10545-018-0178-z)
Supplement: Supplementary file 1 — (DOCX 33 kb) [file 10545_2018_178_MOESM1_ESM.docx]

**Supplementary file 1 – Two acute porphyria questionnaires**

**1. Acute Porphyria Questionnaire**

Completing this form wil take about 15 minutes. Take all the time you need to read everything carefully; try to be as complete as possible when filling out this form.

*(to enter by physician):****PID:*** *________________________* ***Family no.:*** *________________________*

1. **Personal Information and family history**

Surname: ____________________________________________________
Initials: ____________________________________________________
Date of birth: _____________________
Country of birth: ____________________________________________________
Country of residence: ____________________________________________________

Name father: ____________________________________________________
Date of birth father: _____________________
Date of death father: _____________________
Has Porphyria: Yes 🞎 No 🞎 Unknown 🞎

Name mother: _____________________
Date of birth mother: _____________________
Date of death mother: _____________________
Has Porphyria Yes 🞎 No 🞎 Unknown 🞎

Relationship type: 🞎 Single
 🞎 In a relationship / Living together
 🞎 Married
 🞎 Divorced
 🞎 Partner deceased

Do you have children? Yes 🞎 No 🞎 Has porphyria?

Date of birth child 1 ______________________ Yes 🞎 No 🞎
Date of birth child 2 ______________________ Yes 🞎 No 🞎
Date of birth child 3 ______________________ Yes 🞎 No 🞎
Date of birth child 4 ______________________ Yes 🞎 No 🞎
Date of birth child 5 ______________________ Yes 🞎 No 🞎

1. **Questions on your acute porphyria**

In what year did you first experience symptoms of acute porphyria? _______ 🞎 No symptoms
In what year was porphyria diagnosed? _______
How many acute attacks did you experience? _______
How many times were you hospitalized for acute attacks? _______
How many times have you been treated with heme infusions? _______

What symptoms do you experience in relation to your acute porphyria?
🞎 Chronic fatigue 🞎 Headache
🞎 Nausea 🞎 Abdominal pain
🞎 Vomiting 🞎 Back pain
🞎 Diarrhea 🞎 Pain in the arms
🞎 Constipation 🞎 Pain in the legs
🞎 Light-oversensitivity 🞎 Pain in the genital area
🞎 Dark urine
🞎 Incontinence

🞎 Epileptic seizures
🞎 Feeling of ‘pins and needles ‘ (paresthesias) in the extremities
🞎 Muscle weakness or paralysis If YES, where: __________________________
 __________________________
 __________________________

🞎 Anxiety 🞎 High blood pressure
🞎 Depression 🞎 Kidney problems
🞎 Insomnia
🞎 Psychosis, or hallucinations
🞎 Suicidal thoughts

🞎 Respiratory problems
🞎 Coma

If you suffer from attacks, or complaints: What are the precipitating factors? (check all that apply)
🞎 Menstrual cycle-related
🞎 After surgery
🞎 Diet, or fasting
🞎 Alcohol use
🞎 Medication use
🞎 Psychological stress
🞎 Unknown

1. **Other illnesses and lifestyle**

If you have been treated, or seen by a physician, for other ailments than porphyria, please note those here. Hospitalizations, or operations, for reasons other than porphyria should also be listed here.

Medical history Year
1. ____________________________________ _____
2. ____________________________________ _____
3. ____________________________________ _____
4. ____________________________________ _____
5. ____________________________________ _____
6. ____________________________________ _____
7. ____________________________________ _____
8. ____________________________________ _____
9. ____________________________________ _____
10. ____________________________________ _____

Do you smoke? Yes 🞎 No 🞎 Quit smoking 🞎
How much do/did you smoke? ____________________________________________
Age at which you started smoking? ______ years
Age at which you quit smoking? ______ years

Do you drink alcohol? Yes 🞎 No 🞎
How many units per week? < 1 🞎
 1-5 🞎
 6-10 🞎
 11-20 🞎
 >20 🞎

Do you follow a diet? Yes 🞎 No 🞎
If yes, what kind of diet? ____________________________________________
Do you feel limited in life by your Yes 🞎 No 🞎
porphyria?
If yes, please provide a short ____________________________________________
description why you feel ____________________________________________
limited: ____________________________________________

**2 Qualitative Questionnaire Acute Porphyrias – version 1.0, September 2014**

Provide answers in your own words and give a description of what is applicable to your personal situation. With this questionnaire we want to examine what factors are most important for patients with acute porphyria.

Name: ___________________________ PID: _________________________ (to enter by physician)

Porphyria type: [ ] acute intermittent porphyria
 [ ] variegate porphyria
 [ ] hereditairy coproporphyria
 [ ] _______________________ (other porphyrias)

1. What complaints (physically and mentally) do you experience and what factors contribute most to disabilities you experience in relation to your porphyria.

________________________________________________________________________________________________________________________________________________________________________________________________________________________

2. What limitations do you feel you experience due to your porphyria? Due to what complaints?

__________________________________________________________________________________________________________________________________________________________________________________________________________________

3. What complaint could/should we try to resolve to improve your situation? Please give a short explanation if possible.

_____________________________________________________________________________________________________________________________________________________________________________________________________________________
