# Supplementary material for: Medical and financial burden of acute intermittent porphyria
Source: J Inherit Metab Dis. 2018 Apr 19;41(5):809–17. doi: 10.1007/s10545-018-0178-z (PMC6133185; doi:10.1007/s10545-018-0178-z)
Supplement: Supplementary file 2 — (DOCX 17 kb) [file 10545_2018_178_MOESM2_ESM.docx]

**Supplementary file 2 - Detailed methods**

*Definitions of parameters*

Unemployment was defined as a period in which the patient was unable to find or keep a job, due to acute porphyria related symptoms; this is presented with life-long incidence rates. In patients of the school-attending age and students, unemployment was given when there was an inability to keep up progress.

BMI was calculated using the first available BMI measurement, after the age of 18 years. Hypertension was defined as a blood pressure over 140/80 mmHg for longer than 6 months or the use of anti-hypertensive drugs. Chronic kidney disease was defined as an estimated glomerular filtration rate (eGFR) under 60 ml/min existing more than 6 months. The diagnosis of hepatocellular carcinoma was based on histopathological findings.

Anemia was defined as a serum hemoglobin of lower than 8.6 mmol/l that was not associated with acute blood loss. Epilepsy was defined as only epileptic attacks associated with an attack of AIP. Hyponatremia was defined as a serum sodium of lower than 136 mmol/l during a confirmed acute attack.

*Porphyrins, porphyrin precursors and enzyme analyses*

In all subjects porphyrins and precursors were assayed, as described previously(de Rooij et al 2003). In short, delta-aminolaevulinic acid (ALA) and porphobilinogen (PBG) in urine were separated by ion-exchange chromatography and measured spectrophotometrically after condensation with Ehrlich’s reagent (normal values: urine ALA: < 46 μmol/l;
urine PBG: < 9 μmol/l). Plasma ALA and PBG concentrations were measured by a fluorimetric enzymatic assay (normal values: plasma ALA: < 74 nmol/l; plasma PBG: < 12 nmol/l). PBGd (porphobilinogen deaminase) enzyme activity was measured in erythrocyte lysates after incubation with PBG (normal value: < 64 pmol per mg protein per hour). For each individual the first available biochemical test results is presented as baseline characteristic. For PBGd enzyme activity the lowest ever measurement is presented. The reference level for decreased PBGd enzyme activity was < 64 pmol/mg protein/hour measured in erythrocytes.

*DNA analysis*

Genomic DNA was isolated from peripheral blood using the QIAamp DNA Blood Midi Kit (Qiagen) according to the manufacturer’s protocol. The nucleotide sequence analysis of the HMBS gene *(NM_000190.3)* was performed, after PCR, on an Applied Biosystems 3500 Genetic Analyser using the appropriate chemistry.

*Prices used for cost analysis*

The following prices were used to calculate costs: basic costs of a regular hospital admission day were €500. Basic costs for a daycare facility admission were €430 per day. The drug costs of heme therapy was € 731.90 per administration, based on €651.90 per heme arginate ampule and €80 for the albumin (bottle 100ml, concentration 200g/l; heme arginate is best dissolved in albumin).

**REFERENCE**

Rooij FWM de, Edixhoven A, Wilson JHP (2003) Porphyria: a diagnostic approach. T*he Porphyrin Handbook*: Elsevier Science, 212-245.
