# Supplementary material for: Medical and financial burden of acute intermittent porphyria
Source: J Inherit Metab Dis. 2018 Apr 19;41(5):809–17. doi: 10.1007/s10545-018-0178-z (PMC6133185; doi:10.1007/s10545-018-0178-z)
Supplement: Supplementary file 3 — (DOCX 13 kb) [file 10545_2018_178_MOESM3_ESM.docx]

| Recurrent cases (n = 11) | | | Symptomatic cases (n = 24) | | | Asymptomatic cases (n = 53) | | |
| --- | --- | --- | --- | --- | --- | --- | --- | --- |
| Nucleotide change | Mutation  type | # in group | Nucleotide change | Mutation  type | # in group | Nucleotide change | Mutation  type | # in group |
| c.91G>A  c.181dupG  c.346C>T  c.500G>A  *c.160+4A>G | Missense Small Insertion  Missense Missense  Splice site | 2 1  4 3         1 | c.91G>A  c.181dupG  c.219-220delGA c.346C>T  c.500G>A  c.517C>T  c.518G>A  c.973C>T  c.33+1G>A  *c.87+5dupG  c.104C>T  *c.230T>C c.287C>T  c.913-2A>G  *c.1001delT  **Mutation unknown** | Missense Small Insertion Small Deletion Missense Missense Missense  Missense Nonsense   Splice site  Unknown Missense  Missense Missense  Splice site  Small deletion | 2  1 1 5 1 1  1  1  1 1 1  1 1  1  1  **3** | c.91G>A  c.181dupG  c.219-220delGA c.346C>T c.500G>A  c.517C>T  c.518G>A  c.973C>T    c.138C>A  *c.230T>C  *c.602G>C  c.652-1G>C c.667G>A  c.739T>C  c.825+1G>A c.912+1G>A *c.991G>A  **Mutation unknown** | Missense Small Insertion Small Deletion Missense Missense  Missense  Missense Nonsense  Nonsense  Missense  Missense  Splice site Missense Missense Spice site Splice site Missense | 2 5 3 12 3  1  1 1  1  2  2  1  3 1 3  1 2  **8** |

Supplementary file 3 – *HMBS* Mutations in the Dutch cohort

Abbrevations used. HMBS, Hydroxymethylbilane Synthase.

*: unpublished mutation
