# Supplementary material for: Medical and financial burden of acute intermittent porphyria
Source: J Inherit Metab Dis. 2018 Apr 19;41(5):809–17. doi: 10.1007/s10545-018-0178-z (PMC6133185; doi:10.1007/s10545-018-0178-z)
Supplement: Supplementary file 4 — (DOCX 17.1 kb) [file 10545_2018_178_MOESM4_ESM.docx]

**Supplementary file 4 -
Table with baseline biochemical analysis results and enzyme activity in acute intermittent porphyria cohort**

|  | Recurrent  cases  (*n* = 11) | Symptomatic  cases  (*n* = 24) | Asymptomatic  controls  (*n* = 53) | *Normal reference* |
| --- | --- | --- | --- | --- |
| Urine  ALA (µmol/l)  PBG (µmol/l) | 284 (10 – 1740)  356 (10 – 1676) | 109 (7 – 340)  199 (7 – 908) | 34 (5 – 442)  25 (1 – 908) | *< 46 μmol/L*  *< 9 μmol/L* |
| Plasma  ALA (nmol/l)  PBG (nmol/l) | 240 (80 – 2380)  1755 (79 – 6496) | 858 (27 – 2700)  2279 (214 – 6814) | 228 (29 – 2980)  554 (8 – 3846) | *< 74 nmol/L*  *< 12 nmol/L* |
| PBGd enzyme activity*  (pmol/mg/hour) | 51 (34 – 71) | 55 (34 – 98) | 52 (31 – 75) | *> 63 pmol/mg protein/hour* |

Used abbreviations ALA, delta-aminolevulinic acid; PBG, Porphobilinogen; PBGd, porphobilinogen deaminase

All measurements are presented as median (range), and were the first available measurements in a patient.

*presented is the lowest erythrocyte PBGd activity measured.
