# Supplementary material for: Medical and financial burden of acute intermittent porphyria
Source: J Inherit Metab Dis. 2018 Apr 19;41(5):809–17. doi: 10.1007/s10545-018-0178-z (PMC6133185; doi:10.1007/s10545-018-0178-z)
Supplement: Supplementary file 5 — (DOCX 16 kb) [file 10545_2018_178_MOESM5_ESM.docx]

**Supplementary file 5 – Prevalence percentages of all scored porphyric symptoms and complications in the presented AIP cohort**

|  | Recurrent  Cases (n=11) | Symptomatic  cases (n=24) | Asymptomatic controls  (n= 53) | p-value  (R vs S) | p-value  (R vs A) | p-value  (S vs A) |
| --- | --- | --- | --- | --- | --- | --- |
| **Acute % Yes**  **symptoms*** | 100% | 83.3% | 35.8% | 0.535 | p < 0.001 | p < 0.001 |
| Malaise | 81.8% | 29.2% | 1.9% | p = 0.010 | p < 0.001 | p = 0.001 |
| Fatigue | 90.9% | 37.5% | 20.8% | p = 0.009 | p < 0.001 | p = 0.094 |
| Nausea | 100% | 45.8% | 9.4% | p = 0.005 | p < 0.001 | p < 0.001 |
| Vomitting | 90.9% | 58.3% | 9.4% | p = .212 | p < 0.001 | p < 0.001 |
| Diarrhoea | 18.2% | 12.5% | 1.9% | p = 1 | p = 0.076 | p = 0.076 |
| Constipation | 63.6% | 45.8% | 9.4% | p = .712 | p < 0.001 | p < 0.001 |
| Red Urine | 54.5% | 50% | 13.2% | p = 1 | p = 0.007 | p = 0.001 |
| Photosensitivity | 0% | 0% | 1.9% | n/a | n/a | n/a |
| **Pain % Yes**  **symptoms*** | **100%** | **91.7%** | **30.2%** | n/a | p < 0.001 | p < 0.001 |
| Headaches | 36.4% | 29.2% | 13.2% | p =1 | p = 0.089 | p = 0.102 |
| Chest | 9.1% | 4.2% | 1.9% | p =1 | p = 0.321 | p = 0.509 |
| Back | 45.5% | 33.3% | 7.5% | p =0.714 | p = 0.006 | p = 0.004 |
| Abdomen | 90.9% | 79.2% | 28.3% | p =1 | p < 0.001 | p < 0.001 |
| Upper Extremities | 36.4% | 25% | 3.8% | p = 0.696 | p = 0.007 | p = 0.007 |
| Lower Extremities | 45.5% | 25% | 5.7% | p = 0.437 | p = 0.003 | p = 0.017 |
| Genitalia | 0% | 8.3% | 0% | p = 0.542 | n/a | p = 0.086 |
| **Neurological % Yes**  **symptoms*** | **81.8%** | **45.8%** | **17.0%** | p = 0.278 | p = 0.001 | p = 0.008 |
| Paraesthesias | 36.4% | 8.3% | 7.5% | p = 0.146 | p = 0.026 | p = 1 |
| Motor weakness | 45.5% | 20.8% | 7.5% | p = 0.240 | p = 0.006 | p = 0.115 |
| Paralysis | 9.1% | 20.8% | 1.9% | p = 0.657 | p = 0.321 | p = 0.008 |
| Urine incontinence | 0% | 4.2% | 0% | p = 1 | n/a | p = 0.297 |
| Advanced Neuropathy / Coma / Respiratory Failure | 27.3% | 20.8% | 0% | p = 1 | p = 0.004 | p = 0.002 |
| **Psychiatric % Yes**  **symptoms*** | **81.8%** | **33.3%** | **18.9%** | p = 0.012 | p < 0.001 | p = 0.244 |
| Anxiety | 45.5% | 20.8% | 5.7% | p = 0.227 | p = 0.004 | p = 0.099 |
| Depression | 36.4% | 12.5% | 9.4% | p = 0.171 | p = 0.040 | p = 0.699 |
| Psychosis/Hallucinations | 36.4% | 4.2% | 9.4% | p = 0.026 | p = 0.040 | p = 0.659 |
| Insomnia | 27.3% | 20.8% | 11.3% | p = 0.685 | p = 0.177 | p = 0.303 |
| Suicidality | 18.2% | 0% | 1.9% | p = 0.092 | p = 0.074 | p =1 |
|  |  |  |  |  |  |  |
|  | Recurrent  Cases (n=11) | Symptomatic  Cases  (n=24) | Asymptomatic controls  (n= 53) | p-level  (R vs S) | p-level (R vs A) | p-level  (S vs A) |
| Hypertension \| % Yes | 72.7% | 70.8% | 26.4% | p = 1 | p = 0.006 | p < 0.001 |
| CKD \| % Yes | 63.6% | 45.8% | 13.2% | p = 0.471 | p = 0.001 | p = 0.003 |
| HCC \| % Yes | 9.1% | 8.3% | 1.9% | p = 1 | p = 0.316 | p = 0.228 |
| Hyponatremia % Yes | 72.7% | 37.5% | 0% | p = 0.141 | p <0.001 | p < 0.001 |
| Epilepsy % Yes | 45.5% | 12.5% | 0% | p = 0.047 | p <0.001 | p = 0.099 |
| Anemia \| % Yes | 63.6% | 16.7% | 5.7% | p = 0.077 | p <0.001 | p = 0.087 |

Used abbreviations. A, asymptomatic control group; AIP, acute intermittent porphyria; CKD, Chronic Kidney Disease;
HCC, Hepatocellular Carcinoma; R, recurrent cases group; S, symptomatic cases group;

All P-values are calculated with Chi-squared test - Fishers Exact Test. Symptoms are lifelong incidence in relationship to patients AIP history. Information on symptoms was retrieved from electronical and paper patient charts or self-reported via questionnaires filled in by patients.
