# Supplementary material for: Medical and financial burden of acute intermittent porphyria
Source: J Inherit Metab Dis. 2018 Apr 19;41(5):809–17. doi: 10.1007/s10545-018-0178-z (PMC6133185; doi:10.1007/s10545-018-0178-z)
Supplement: Supplementary file 6 — (DOCX 13 kb) [file 10545_2018_178_MOESM6_ESM.docx]

**Supplementary file 6 - Detailed descriptions of deceased acute intermittent porphyria (AIP) subjects**

The first fatal case was a female patient who had a first porphyric attack at age 36 years, yet recurrent attacks started in her sixth decade of her life. She died at age 71 years, from hepatocellular carcinoma (HCC) related metastatic disease, 3 years after her initial HCC diagnoses and hemi-hepatectomy. HCC was diagnosed as an incidental finding on a routine liver ultrasound for monitoring iron related liver fibrosis. She was not regularly screened for HCC, which is currently the standard care for all acute hepatic porphyria patients over age 50 years.

The second patient was a male patient with recurrent attacks since his third decade of life. He was on weekly prophylactic heme therapy. His medical history included premature cardiovascular disease with coronary bypass and aortic dissection. At age 41 years, he suffered an acute porphyric attack with psychosis, despite prophylactic heme. The porphyric attack was complicated by a traumatic head injury; a skull fracture and severe hemorrhagic contusions in the right frontal lobe, later complicated by seizures. The symptoms progressed to quadriplegia, seizures, and respiratory failure with mechanical ventilation. He died 8 months later, age 42 years, from pulmonary complications of chronic mechanical ventilation.

The third case was a female patient with recurrent attacks since the age of 30 years. She was completely immobilized after several AIP attacks with progressive motor neuropathy and psychiatric exacerbations. Prophylactic heme therapy was unsatisfactory for her symptoms. She suffered from depressive episodes for which she was treated with alprazolam for years: mianserin, and lormetazepam, without sufficient effect on her mood. She repeated requested to end her life. She died at age 34 years following euthanasia, this request was granted following consultation of an independent physician from the Support and Consultation team for Euthanasia in the Netherlands; her situation was considered unbearable suffering without prospects of improvement.

The fourth case was a female patient who was diagnosed with AIP in her thirties. Following several severe attacks, weekly prophylactic heme therapy was started, however, several more porphyric attacks occurred, one attack complicated by convulsions. She suffered from chronic pain, complicated by morphine dependency, complicated by abscesses at injection sites, constipation and a traumatic sigmoid perforation following a self-administered enema. Alternative pain management with nitrous oxide was complicated by self-inflicted overdose leading to temporary coma, anemia and a partly irreversible polyneuropathy. Her medical history mentions gallstones, hypothyroidism, and antiphospholipid syndrome with a vena cava superior syndrome. The anticoagulant therapy was complicated by a non-traumatic subdural hematoma. She was bedridden with a low quality of life. She developed progressive kidney disease with secondary anemia, renal osteodystrophy, and poly-articular gout. The patient was anorexic and malnourished. She died following complications at age 65 years, details are unknown.

The fifth patient was a male patient presenting at age 41 years with his first and only two uncomplicated porphyric attacks. At age 72 years, HCC was diagnosed following an incidental finding on a PET CT-scan performed for headache. He underwent hemi-hepatectomy but died from a brainstem hemorrhage 13 days post-operatively.
